# Quantitative analysis of organizational culture in occupational health research: a theory-based validation in 30 workplaces of the organizational culture profile instrument

**DOI:** 10.1186/1471-2458-13-443

**Published:** 2013-05-04

**Authors:** Alain Marchand, Victor Y Haines, Julie Dextras-Gauthier

**Affiliations:** 1 School of Industrial Relations, University of Montreal, Montreal, Canada; 2 Public Health Research Institute, University of Montreal, Montreal, Canada

## Abstract

**Background:**

This study advances a measurement approach for the study of organizational culture in population-based occupational health research, and tests how different organizational culture types are associated with psychological distress, depression, emotional exhaustion, and well-being.

**Methods:**

Data were collected over a sample of 1,164 employees nested in 30 workplaces. Employees completed the 26-item OCP instrument. Psychological distress was measured with the General Health Questionnaire (12-item); depression with the Beck Depression Inventory (21-item); and emotional exhaustion with five items from the Maslach Burnout Inventory general survey. Exploratory factor analysis evaluated the dimensionality of the OCP scale. Multilevel regression models estimated workplace-level variations, and the contribution of organizational culture factors to mental health and well-being after controlling for gender, age, and living with a partner.

**Results:**

Exploratory factor analysis of OCP items revealed four factors explaining about 75% of the variance, and supported the structure of the Competing Values Framework. Factors were labeled Group, Hierarchical, Rational and Developmental. Cronbach’s alphas were high (0.82-0.89). Multilevel regression analysis suggested that the four culture types varied significantly between workplaces, and correlated with mental health and well-being outcomes. The Group culture type best distinguished between workplaces and had the strongest associations with the outcomes.

**Conclusions:**

This study provides strong support for the use of the OCP scale for measuring organizational culture in population-based occupational health research in a way that is consistent with the Competing Values Framework. The Group organizational culture needs to be considered as a relevant factor in occupational health studies.

## Background

The expansion of occupational health research to better account for a broader set of contextual factors that are associated with occupational stress and strain remains a significant challenge. Scholars in this field are paying more attention to workplace-level factors, but with an almost exclusive focus on specific psychosocial safety climate variables [1,2]. Notwithstanding such recent advances in the area of macro-organizational influences, the bulk of work stress research remains focused on task-level factors like psychological demands and job control [3], or effort and rewards [4]. Although this work design perspective has helped qualify a number of risk factors or stressors that may cause psychological strain and ill-health, it does not account for the broader organizational context in which work is performed.

For occupational health research to progress, researchers can no longer ignore contextual factors nor the functions of organizational culture in the stress process [5]. Organizational culture is formed through meaningful accumulated learning at the organizational level; experiences of success and failure that are retrieved and incorporated into the culture of an organization and shared by the members [6]. Considering that organizational culture is a meaningful social characteristic with potentially significant health consequences [5,7-10], occupational health researchers engaged in population-based investigations across levels of analysis will increasingly need to address the challenge of using a questionnaire measure of organizational culture in a manner that will allow replications and cross-sectional comparative studies within an accepted frame of reference.

Given the usefulness of quantitative measures of organizational culture [11] and the need to integrate this fundamental dimension of the workplace context into occupational stress research, the aim of this study is to advance a measurement approach for the study of organizational culture in population-based occupational health research, and to examine a relatively simple short-form questionnaire measure of organizational culture. In the context of complex multilevel occupational health research, other approaches that involve collecting qualitative data [12,13] or a multi-method conceptualization of organizational culture are considered difficult to apply. We will argue further on that a meritorious alternative for such research may be found either in the 26-item Organizational Culture Profile (OCP) survey instrument [14] or in a shorter form derived from this instrument.

A related challenge is to determine which facets or dimensions of organizational culture are most relevant to the study of work stress and occupational health. Drawing from the Competing Values Framework [15,16], meta-analytic evidence suggests a large significant positive relationship between the Group culture and job satisfaction, whereas the Developmental culture is more strongly positively associated with innovation [17]. Some types of organizational culture therefore appear to be more relevant than others depending upon the phenomenon being addressed by the research. Just as some culture types appear to be well suited to the study of organizational innovations [18] or patient satisfaction [19], or other outcomes of interest, other culture types might more applicable to the study of occupational health. This study will therefore seek to advance a measurement approach that is not only feasible, but also most relevant to occupational stress research. We will therefore seek to determine which culture types are most strongly related to employee mental health and well-being.

This study will therefore validate a measurement approach and test how different organizational culture types are associated with psychological distress, depression, emotional exhaustion, and well-being. By doing so, we hope to a) provide multi-level evidence of the associations between organizational culture and these outcomes of interest and b) offer some guidance to those researchers who wish to include organizational culture in their models and thereby capture some aspects of group dynamics [20]. In the following sections we describe the OCP survey instrument [14] and explain how the Competing Values Framework [15,21] might offer a valuable theoretical backdrop for assessing the construct validity of this measure. We then test the construct validity of the 26-item OCP questionnaire instrument with a sample of 1,164 employees nested in 30 workplaces. Next, we test associations between the dimensions of the OCP survey instrument and employee psychological distress, depression, emotional exhaustion and well-being. This theory-based measurement approach, which is clearly lacking in the analysis of OCP items [22], is intended to contribute to the advancement of occupational health research involving multilevel, population-based investigations that consider the meaningful facets of the organizational context in which stress occurs.

### Organizational culture profile survey instrument

The Organizational Culture Profile (OCP) proposed by O’Reilly et al. (1991) is one of the most widely cited survey instruments in the organizational culture literature. At the time of writing these lines, their published paper was cited 2,431 times (Google Scholar). Although this scale was initially developed to assess person-organization fit [14,23-26], it holds much promise for population-based research seeking to model the effects of organizational culture on group- or individual-level phenomena. Nonetheless, possibly because of its initial focus, little is known about the aggregate-level properties of this measure. Only a few studies have analyzed these properties [27-29] and most of these were conducted in a single industrial sector with small samples of workplaces. Moreover, the approach has so far been inductive rather than theory-based.

Another issue related to the use of this survey instrument in population-based investigations relates to the number of items that it includes. The original measure had 54 items, but was reduced to 26 [14]. Subsequent studies applied either the longer or the shorter forms of the scale. Though the size of the longer scale makes it impractical for large scale population-based studies, the 26-item scale would benefit from further validation that would also review the group-level properties of the measure. A related concern is that factor analysis requires at least 10 to 20 subjects per item to achieve a reliable solution [30] and studies have failed to meet this requirement when using either the 54-item scale [31-34] or the 26-item scale [35,36]. These studies were characterized by overall small employee sample sizes.

There are also considerable inconsistencies in the conceptual structure of the OCP scale. Empirical analysis of the scale items initially generated seven factors labeled innovation, stability, respect to people, outcome orientation, attention to details, team orientation, and aggressiveness [14]. In subsequent studies, the number of factors was reported to be one [31], five [32,36], six [35,37], seven [27-29,34,38], and eight [33]. Factor loadings also varied considerably from one study to another as did the labeling of the factors. New factors like procedural [33], easygoing [33,38], supportive, humanistic, and task orientated [32], teamwork [35], and teamwork/respect for people [36] have shown up from one study to the next. These inconsistencies obviously limit the ability to study occupational health phenomena with a common frame of reference and thereby to accumulate a significant body of knowledge relating specific organizational culture types to psychological distress, depression, emotional exhaustion, and well-being.

Such conceptual and measurement challenges are not surprising given that those involved in developing the original scale were less interested in fitting its structure to some established theory or typology of organizational culture than they were in measuring individuals and organizations along commensurate items (i.e., values) for assessing person-organization fit. Without a guiding theoretical framework for the measurement of organizational culture, inconsistencies in its factorial structure might have been expected. Moreover, with significant advances in multilevel theory and research since the original OCP measure was conceived, considering the additional measurement issues that arise at higher levels of analysis [39], the need to establish its group-level properties could not be overstated; especially when one considers that the proper level of analysis for the study of organizational culture is the organization, unit, or workplace.

### Competing values framework

The only modest theoretical anchor for the OCP scale is that it accepts the premise that values are central components of organizational culture. Founded on this same basic premise, different typologies of organizational culture were developed over time [40-43]; including the highly influential competing values framework [15,21]. Although it is not the only value theory with competing values, as exemplified by the Schwartz’s theory of universals in values [22,44], it is widely applied in studies that address the influence of organizational culture on workplace phenomena [45-51]. From the vantage point of this framework, the culture of an organization is defined along two bipolar dimensions or continuums. The first contrasts an internal with an external focus, thereby opposing integration and unity to differentiation and rivalry. The second opposes the search for stability, order and control to flexibility and change. According to their location on these two continuums, four types of organizational culture are described. The Group culture (internal – flexibility/change) favours employee participation, cooperation, mutual trust, team spirit, learning, fulfilling work through human resource development, trust in human potential, cohesiveness, and synergy. The Hierarchical organizational culture (internal – stability/order/control) is characterized by stability and continuity, information management, division of labour, efficiency, formal procedures, order, control, and rules and regulations. The Developmental culture (external – flexibility/change) relies upon environmental scanning, experimenting, innovating, organizational transformation through organic growth or market acquisitions, learning, creativity, adaptability, and growth. The Rational culture (external – stability/order/control) emphasizes decision rules, performance indicators, individual and collective accountabilities, reinforcement contingencies, production, and achieving goals and objectives.

With its widespread appeal and its focus on values, the Competing Values Framework offers an appropriate theoretical basis for assessing the construct validity of the OCP measure. Moreover, the culture types this framework describes appear like they may have some meaningful interpretations in occupational health research. Indeed, values such as “cooperation” and “mutual trust” that characterize the Group culture apparently reflect the extent or quality of relationships at the workplace level. Previously cited findings showing positive associations between the Group culture and attitudinal outcomes lend further support to the association between this culture type and employee well-being. Other arguments could be made to support the associations between other culture types and employee-level mental health and well-being outcomes [5].

Although this framework advances a measurement approach with the Organizational Culture Assessment Instrument (OCAI), application of this instrument in population based research comprising a diversity of workplaces and employees from different occupations or with varying education levels remains an unrealistic proposition. The OCAI includes six domains with four items to be assessed within each domain. Respondents are prompted to weight each item with a maximum of one hundred points per domain. This makes answering this instrument time consuming and unsuitable for people having low educational attainments as well as for questionnaire surveys that seek to measure several variables at a time.

Table 1 presents the principal values of the Competing Values Framework and the corresponding OCP scale items sorted by the authors according to the above-mentioned four culture types.

**Table 1:** OCP items and organizational culture types according to the competing values framework

**Competing values framework**		**OCP**
**Culture type**	**Principal values**	**OCP scale items**
Group	Cohesion	Fairness (OCP1)
	Morale	Respect for the individual’s rights (OCP2)
	HR development	Tolerance (OCP3)
	Open communication	Being socially responsible (OCP4)
	Cooperation	Being people oriented (OCP10)
	Trust	Being team oriented (OCP11)
	Teamwork	Working in collaboration with others (OCP12)
	Openness	
	Participation	
Hierarchical	Stability order	Being careful (OCP19)
	Rationality	Paying attention to detail (OCP20)
	Predictability	Being precise (OCP21)
	Security	Being rule oriented (OCP22)
	Coordination of activities	Security of employment Stability (OCP23)
	Procedures	Stability (OCP24)
	Streamlined operations	Predictability (OCP26)
Developmental	Flexibility	A willingness to experiment (OCP14)
	Creativity	Not being constrained by many rules (OCP15)
	Experimentation	Being quick to take advantage of opportunities
	Risk	(OCP16)
	Autonomy	Being innovative (OCP17)
	Adaptation	Risk taking (OCP18)
	Readiness	
	Innovative	
Rational	Productivity	Being competitive (OCP5)
	Efficiency	Achievement orientation (OCP6)
	Competitiveness	Having high expectations for performance (OCP7)
	Aggressiveness	Being results oriented (OCP8)
	Results orientation	Being analytical (OCP9)
	Planning and goal setting	Action oriented (OCP13)
	Action oriented	Being aggressive (OCP25)
	Producing outputs	

Assuming that culture varies from one workplace to another [52], there therefore appear to be close ties between the values assigned to the culture types of the Competing Values Framework and those included in the OCP survey instrument.

In sum, the literature has, on the one hand, been abuzz about organizational culture, but also relatively quiet when it comes to empirical demonstration of the construct in population-based multilevel research conducted in the area of occupational health. Even with a steady increase in the number of multilevel research models being tested in this area [1,53,54], there remains an ongoing and questionable disregard for organizational culture. The development of a questionnaire measure for the study of organizational culture in population-based occupational health research would offer a means for the advancement of such models.

## Methods

### Data

Data were collected in 2009–2010 within 30 Canadian workplaces randomly selected from a list of over 500 companies insured by a large insurance company. Those companies randomly selected by the researchers were invited by their insurer to participate in this study and those workplaces that accepted were referred to the research team. The workplaces in our sample were quite diverse in terms of their products, services, and markets (e.g., motor manufacturing, software development, plumbing supplies, airport maintenance), with 18 in manufacturing and 12 in the service sector. Half (15) of the participating workplaces were unionized and they ranged in sizes from 9 to 391 employees, with an average of 96.9 workers per workplace. In each workplace, researchers first sent a communication to inform all employees about the research project. Then, a random sample of employees was selected and they were invited by the researchers to individually complete a questionnaire on company time using a touch-screen monitor. Consenting workers signed an informed consent and were given the necessary instructions. The questionnaire covered several aspects related to health and well-being, work, family, neighborhood, social networks, personality traits, and demographics. Questionnaire items related to the present study are provided as an Additional file. Overall, 1,164 employees agreed to participate in the survey, for a response rate of 72.5%. Workplace response rates ranged from 51.2% to 100%. On average, 38.8 of employees per workplace completed the questionnaire (minimum = 8; maximum = 136); 31.3% were female, the mean age was 39.3 (*SD*=10.4), and 69.9% where living in a couple. After deleting cases with missing values, that available workers sample size was n=1153. The study protocol was approved by the Ethical Committees of the University of Montreal, McGill University, Laval University and Bishop’s University.

### Measures

#### Organizational culture

All respondents completed the 26-item OCP scale [14] in either English or French. This scale includes as many values descriptive of organizational culture (e.g., fairness, risk taking). Respondents were prompted to indicate to what extent each of the values listed in the scale describes their organization on a unipolar rating scale ranging from *not at all* (1) to *a great extent* (5).

#### Mental health and well-being

Psychological distress was measured with the General Health Questionnaire (GHQ) short-form, 12-item scale [55] (alpha=0.85) (e.g., *unable to concentrate on whatever you’re doing, feeling constantly under strain*), and depression with the Beck Depression Inventory (BDI) 21-item scale [56] (alpha=0.91) (e.g., *feeling sad, suicide thoughts*). Using available cut-points for psychological and depression, 23.6% of workers were reporting feeling of distress and 5.7% were reporting moderate to severe symptoms of depression. Emotional exhaustion was assessed with five items from the Maslach Burnout Inventory (MBI) 16-item general survey [57] (alpha=0.90) (e.g., *feeling emotionally drained from work, feeling used up at the end of the workday*), and well-being was measured with the five-item WHO Well-Being Index (WHO-5) [58] (alpha = 0.83) (e.g., *feeling cheerful and in good spirits, feeling calm and relaxed*).

#### Control variables

Gender was measured as either male (0) or female (1). Age was measured in years and a value of 0 was assigned to respondents not living with a partner and of 1 if living with a partner (i.e., couple). Employment income was measured by asking each respondent to report his or her salary, before taxes and deductions for the past 12 months on a 10-point ordinal scale ranging from 1 = Less than $20,000 to 10 = $100,000 or more. Education level was assessed as the highest diploma obtained on a 10-point ordinal scale ranging from 1= No diploma to 10 = Doctoral. Working hours were measured by the number of hours spent on the job, and Work schedule irregularity was measured on a 4-point Likert-type scale (never/all the time) addressing the frequency of the respondent’s exposure to irregular or unpredictable schedules.

### Analysis

Exploratory factor analysis was conducted to assess the dimensionality of the OCP scale. Factors were extracted using the iterated principal-factor method on the correlation matrix. The number of retained factors was based on the Horn’s Parallel Analysis Test [59]. Oblique rotation with Kaiser Normalization was applied to extract factors loadings because the factors were expected to correlate as multiple perceptions of organizational culture may coexist within the same organization [40,60-62]. Scale reliabilities (Cronbach alphas) were computed with items retained from the rotated factor structure. Multilevel models, with workers (level-1) nested in their respective workplace (level-2), were estimated with restricted maximum likelihood to assess workplace-level variations. If organizational culture exists as a contextual component of workplaces, significant variations of rotated factors are expected at the workplace level. Intraclass correlations quantified the magnitude of these between workplace variations. Finally, multilevel regression models were estimated to evaluate the contribution of organizational culture factors to mental health and well-being after controlling for gender, age, and living with a partner. These three control variables were retained because they were routinely associated with the outcomes [63-65].

## Results

Table 2 presents the descriptive statistics and correlations for all the scale items. The correlation coefficients between the 26 OCP scale items ranged from -.20 to.74, with an average correlation of *r* =.28. It should be notes that the correlations between OCP15 and the other scale items were generally negative.

**Table 2:** Descriptive statistics and correlations for the OCP scale items

	**Mean**	**SD**																									
OCP1	3.44	0.93																									
OCP2	3.89	0.85	.65																								
OCP3	3.76	0.81	.45	.61																							
OCP4	3.78	0.77	.50	.61	.57																						
OCP5	3.86	0.88	.25	.28	.26	.36																					
OCP6	4.01	0.78	.27	.29	.27	.38	.64																				
OCP7	3.89	0.79	.08	.11	.09	.23	.48	.58																			
OCP8	3.99	0.74	.13	.15	.11	.20	.45	.58	.66																		
OCP9	3.60	0.85	.19	.14	.12	.23	.32	.39	.43	.48																	
OCP10	3.42	0.99	.56	.59	.44	.52	.24	.30	.12	.17	.26																
OCP11	3.63	0.96	.47	.49	.40	.47	.29	.37	.19	.26	.30	.62															
OCP12	3.71	0.85	.48	.54	.44	.51	.25	.32	.18	.17	.23	.62	.74														
OCP13	3.65	0.79	.36	.34	.27	.38	.39	.44	.38	.37	.30	.39	.42	.46													
OCP14	3.41	0.93	.32	.30	.26	.37	.26	.28	.17	.15	.27	.42	.44	.47	.48												
OCP15	2.26	1.24	-.20	-.16	-.11	-.11	-.07	-.06	.02	-.02	-.03	-.12	-.08	-.08	-.05	.01											
OCP16	3.38	0.87	.29	.27	.19	.34	.37	.40	.30	.29	.24	.36	.37	.36	.46	.47	.11										
OCP17	3.47	0.92	.33	.31	.24	.42	.37	.39	.26	.26	.34	.38	.43	.41	.45	.58	.03	.56									
OCP18	2.94	0.92	.17	.15	.18	.23	.23	.23	.18	.16	.19	.22	.23	.25	.31	.48	.13	.42	.50								
OCP19	3.60	0.78	.16	.25	.17	.23	.09	.17	.19	.15	.12	.29	.22	.26	.16	.11	.07	.08	.09	-.11							
OCP20	3.56	0.82	.38	.38	.23	.39	.24	.29	.22	.21	.31	.41	.41	.44	.35	.36	-.04	.35	.39	.19	.37						
OCP21	3.59	0.80	.40	.39	.27	.41	.23	.32	.21	.22	.32	.45	.45	.50	.39	.41	-.02	.39	.43	.22	.35	.71					
OCP22	3.69	0.81	.28	.35	.24	.36	.21	.28	.27	.26	.28	.35	.36	.36	.31	.21	-.12	.17	.23	.04	.42	.47	.48				
OCP23	3.62	0.90	.33	.40	.31	.38	.16	.22	.09	.09	.12	.44	.39	.45	.23	.27	.00	.30	.26	.10	.31	.34	.38	.36			
OCP24	3.69	0.80	.39	.44	.33	.39	.23	.27	.11	.14	.15	.48	.40	.46	.29	.32	-.02	.31	.29	.15	.31	.41	.43	.36	.73		
OCP25	2.65	1.11	.02	-.03	.02	.05	.24	.22	.21	.23	.13	.01	.06	.01	.23	.07	.02	.18	.18	.21	-.09	.00	.04	.03	-.06	.00	
OCP26	3.20	0.85	.19	.28	.19	.24	.19	.21	.19	.17	.23	.27	.23	.28	.27	.19	.01	.22	.22	.11	.26	.24	.29	.32	.29	.31	.21

The rotated solution from the factor analysis is presented in Table 3 along with item Squared Multiple Correlation (SMC). Based on Horn’s Parallel Analysis Test, four factors are identified that together account for about 75% of the scale variance.

**Table 3:** Results of exploratory factor analysis with oblique rotation

**Items**	**Factor 1**	**Factor 2**	**Factor 3**	**Factor 4**	**SMC**
Fairness (OCP1)	.68	.03	.06	-.02	.51
Respect for the individual’s rights (OCP2)	.86	.03	-.08	.00	.71
Tolerance (OCP3)	.74	-.10	-.03	.03	.48
Being socially responsible (OCP4)	.63	.05	.09	.13	.55
Being competitive (OCP5)	.23	-.13	.11	.60	.49
Achievement orientation (OCP6)	.19	-.03	.07	.69	.63
Having high expectations for performance (OCP7)	-.11	.07	-.01	.81	.64
Being results oriented (OCP8)	-.04	.04	-.03	.80	.62
Being analytical (OCP9)	-.06	.17	.14	.44	.32
Being people oriented (OCP10)	.56	.22	.16	-.04	.59
Being team oriented (OCP11)	.44	.20	.23	.05	.53
Working in collaboration with others (OCP12)	.47	.26	.24	-.04	.59
Action oriented (OCP13)	.17	.08	.35	.30	.45
A willingness to experiment (OCP14)	.08	.11	.67	-.06	.55
Not being constrained by many rules (OCP15)	-.34	.11	.22	-.06	.09
Being quick to take advantage of opportunities (OCP16)	.00	.09	.59	.15	.49
Being innovative (OCP17)	.05	.07	.68	.11	.61
Risk taking (OCP18)	-.03	-.15	.73	.02	.47
Being careful (OCP19)	.01	.62	-.23	.09	.37
Paying attention to detail (OCP20)	.01	.62	.18	.05	.52
Being precise (OCP21)	.01	.63	.25	.03	.59
Being rule oriented (OCP22)	.10	.58	-.14	.20	.46
Security of employment Stability (OCP23)	.25	.50	.04	-.10	.43
Stability (OCP24)	.27	.48	.07	-.06	.46
Being aggressive (OCP25)	-.07	-.16	.21	.30	.15
Predictability (OCP26)	.07	.31	.05	.14	.19
Variance explained	.47	.13	.08	.07	
Factor correlations	1.00				
	.56	1.00			
	.48	.47	1.00		
	.32	.37	.51	1.00	

Low SMC coefficients are manifest for items OCP15 (.09), OCP25 (.15), and OCP26 (.19), suggesting a small contribution of these items to the factorial solution. Most items did, however, load as expected on their predicted dimensions (see Table 1) and the factor correlations support the assumption of correlated factors. It is also quite clear that there is a strong correspondence between the factorial structure obtained and the Competing Values Framework. Consistent with the expectation expressed in Table 1, the first factor reflects the Group culture; the second, the Hierarchical culture; the third, the Developmental culture; and the fourth, the Rational culture. Only OCP13 and OCP15 deviate from their expected factors. OCP13 (*action oriented*) was expected to load on the Rational culture dimension, but the results indicate a better contribution to the Developmental culture. OCP15 (*not being constrained by many rules*) was expected to load on the Developmental culture; but the results indicate a better contribution to the Group culture. OCP13 was then included as an item in the Developmental culture and OC15 was added to the Group culture. However, because the factor loading of OCP15, OCP25 and OCP26 were low, they were removed in the following analysis. Factor reliabilities based on Cronbach’s alpha (α) are quite acceptable with an alpha of .89 for the Group culture, an alpha of .82 for both the Hierarchical and the Developmental culture types, and an alpha coefficient of .83 for the Rational culture.

The following analysis examined between-workplace variations in the Group, Hierarchical, Developmental, and Rational culture types. The results of the multilevel linear regression models are displayed in Table 4.

**Table 4:** Multilevel analysis of organizational culture types

	**Group**	**Hierarchical**	**Developmental**	**Rational**
Scale range	7-35	6-30	5-25	5-25
Mean	25.44	21.59	16.78	19.33
SE	0.38	0.19	0.21	0.14
σ^2^_workplaces_	3.55	0.67	0.93	0.24
σ^2^_workers_	21.03	13.52	11.43	10.23
χ^2^ (σ^2^_workplaces_ = 0)	74.45*	28.24*	53.91*	9.11*
Rho	.14	.05	.08	.03
* p <0.01				

These results show that the four types of organizational culture varied significantly between the 30 workplaces. Moreover, intraclass correlations (Rho) ranged from .03 to .14, expressing that 3% to 14% of the scale variance are between workplaces. The Group culture appears to show the larger differences across workplaces compared to the other culture types.

Finally, considering our interest in occupational health, we examined whether the four organizational culture types were associated with mental health and well-being outcomes. This can be considered a test of the nomological validity of the culture types generated from the above analyses. As a form of construct validity, nomological validity offers a test of the degree to which the dimensions of the construct behave as expected within a system of related constructs.

Before including variables in the models, a set of analysis were carried out to evaluate if the four outcomes varied between workplaces. Results revealed significant between workplace variations for psychological distress (χ^2^=26.51 df 1 p<.01; rho=0.02), depression (χ^2^=3.96 df 1 p<.05; rho=0.02), emotional exhaustion (χ^2^=27.74 df 1 p<.01; rho=0.05), and well-being (χ^2^=29.73 df 1 p<.01; rho=0.05), which supported the adequacy of the use of further multilevel modelling. After controlling for gender, age, marital status, employment income, education level, work hours per week and irregular work schedule, results in Table 5 indicate that the Group culture types is negatively associated with psychological distress, while the Rational culture type is associated with elevated higher level of psychological distress. The same pattern is observed for depression, with the exception of a non-significant association for the Developmental culture type. As for emotional exhaustion, the same variables play the same way as what it was observed for psychological distress. In the fourth regression model, Group and Hierarchical culture types are related to higher scores on well-being. Overall, the Group organizational culture type is consistently and more strongly associated with these mental health and well-being outcomes compared to the other types of organizational culture. With the exception of well-being, the Hierarchical culture appears to be poorly related to these outcomes. It would appear that the greatest contrasts in these patterns of results are between the Group and the Rational culture types. The Group culture is associated with positive health outcomes whereas the Rational culture is quite consistently associated with negative health outcomes.

**Table 5:** Mutilevel analysis of the relationship between organizational culture, psychological distress, depression, burnout, and well-being

	**Distress**		**Depression**		**Emotional exhaustion**		**Well-being**	
	**Estimate**	**Z**	**Estimate**	**Z**	**Estimate**	**Z**	**Estimate**	**Z**
Group	-0.10**	-4.83	-0.44**	-7.86	-0.09**	-8.72	0.22**	5.60
Hierarchical	-0.02	-0.83	0.02	0.23	-0.02	-1.35	0.13**	2.78
Developmental	-0.05	-1.90	-0.13	-1.78	-0.04**	-2.74	-0.09	1.71
Rational	0.07*	2.45	0.21**	2.83	0.08**	5.69	-0.02	-0.35
Gender (female)	0.75**	4.28	2.07**	4.52	0.35**	4.29	-0.76*	-2.36
Age	-0.02**	-2.78	-0.08**	-3.95	-0.02**	-4.29	0.06**	4.55
Couple (partner)	-0.36*	-2.12	-1.12*	-2.53	0.04	0.49	0.54	1.82
Employment income	0.01	0.23	-0.07	-0.59	0.00	0.02	0.12	1.34
Education	-0.07	-1.52	-0.39**	-3.34	-0.05*	-2.45	0.08	0.97
Work hours (per week)	0.02	1.79	0.03	0.66	0.01	1.19	-0.06*	1.99
Irregular work schedule	0.13	1.31	0.58*	2.22	0.27**	5.57	-0.34	-1.87
Constant	4.57**	5.15	19.31**	8.35	3.37**	7.91	5.36**	3.34
Random part								
Variance (firms)	.041		.35		.033**		1.05**	
Variance (workers)	6.25**		42.12**		1.49**		19.45**	
Model fit								
X^2^ (11 df)	105.16**		199.88.84**		267.96**		185.16**	

* p <.05, ** p<.01.

As far as the covariates are concerned, gender and age are systematically related to the four outcomes, while being in couple is associated with psychological distress and depression. Employment income is unrelated to mental health and well-being, while education level is associated negatively with psychological distress and depression. The number of work hours per week is related to lower feelings of well-being, and having an irregular work schedule is associated with more feelings of depression and emotional exhaustion. Finally, Table 5 shows that psychological distress and depression do not vary across firms after including the organizational culture types and control variables, while emotional exhaustion and well-being still vary significantly between firms.

## Discussion

Although an extensive literature has developed on the topic of organizational culture, few studies were devoted to its measurement and to its association with workers mental health and well-being outcomes. This study assessed the construct validity and workplace-level properties of the 26-item OCP instrument [14] and its association with mental health and well-being outcomes. The findings support the proposition that the OCP measure can be conceptualized in terms of the four dimensions of the Competing Values Framework [15,21]. This validation also gives a clear cut decision rule for the number of factors derived from the OCP. The findings also show that the workplace-level properties of the OCP scale are adequate, suggesting that it could be applied at the group level in future multilevel studies. The associations between the culture types derived from this scale and mental-health and well-being outcomes further underscore the need to consider organizational culture in occupational health research.

With the exception of two items, the distribution of the OCP items according to organizational culture types is consistent with the Competing Values Framework. Three items (OCP15, OCP25, OCP26), however, had low loadings and were removed from the scale. Overall, seven items loaded on the group culture (OCP1-OCP4, OCP10-OCP12), six on hierarchical type (OCP19-OCP24), five on the developmental type (OCP13,OCP14, OCP16-OCP18), and five items loaded on the rational type of culture (OCP5-OCP9). Table 6 in Appendix 1 describes the final items distribution for each culture type. Furthermore, the four-factor model is responsive to the need for parsimony as well as the need for plausibility in the number of factors selected [66]. A solution with four common factors is obviously more parsimonious than models with seven or eight factors. Moreover, with reference to the Competing Values Framework, the four-factor model has a sufficient number of factors to account for the “major” factors that define organizational culture across companies and economic sectors [28,34,36]. Drawing from a heterogeneous sample of workplaces, our analysis suggests that four factors are sufficient to capture the essential aspects of organizational culture. This measurement approach has the added benefit of being theoretically grounded as it is associated with clear conceptual definitions of different culture types [15,21]. The Competing Values Framework seemingly provides clear conceptual definitions of the Group, Hierarchical, Developmental, and Rational culture types that contrast integration and unity to differentiation and rivalry, and that also oppose the search for stability, order and control to flexibility and change.

Our findings also support a measurement approach that is based on employee perceptions as they seemingly distinguish culture types between firms. This finding is important for advancing multilevel research that generally aggregates such perceptions to reflect unit-level phenomena. Because organizational culture is assumed to be a contextual, firm-level construct, then it must vary between firms or workplace. The results we obtained using multilevel regression modeling show significant variations of all four organizational culture types between workplaces. However, larger differences between workplaces were observed for the Group culture compared to the Hierarchical, Developmental, and Rational cultures. This may indicate that the Group culture is better suited for distinguishing of organizations compared to other types of organizational culture. For the other culture types, we did observe significant differences between workplaces but these differences were not as striking. Therefore, according to the results reported here, workplaces are not important determinants of hierarchical, developmental and rational organizational culture types.

After controlling for employment income, education level, number of work hours per week, and irregular work schedules, we found the Group organizational culture type to be consistently and moderately correlated with mental health and well-being outcomes. Overall, the Group organizational culture performs better at distinguishing organizations compared to other types of organizational culture and this dimension had the strongest associations with mental health and well-being outcomes. Such findings support the use of the Group organizational culture as a significant factor for the study of occupational health. Our results indicate that it can be adequately and conveniently measured with seven items from the OCP instrument. The internal consistency of the Group culture was high. Because the Group organizational culture puts a strong emphasis on human resources and promotes a supportive work environment, employees in organizations characterized by this culture type might experience less work stress that may explain why they seem to report fewer mental health problems and higher levels of well-being.

We therefore recommend that the seven-item Group culture scale be used in future population-based occupational health studies that operate across levels of analysis and that aim to offer a more complete account of the contextual factors that are directly or indirectly associated with occupational health outcomes. In a previous study, the Group culture was associated with employee satisfaction [17] and our findings underscore its relevance for the study of occupational health. Given that the aim of much occupational health research is to identify risk factors in the work context rather than sort workplaces according to culture types, this measurement approach may be the most responsive to the actual needs of researchers. By addressing the measurement challenge in such a way, scholars might therefore gain valuable insights into a meaningful contextual factor. Our findings further suggest that they may do so while avoiding the significant complexities of other measurement approaches that involve measuring a wider range of culture types.

This study has some limitations. First, this is a cross-sectional study which implies that the relationships observed cannot be interpreted causally and will need to be replicated longitudinally. It is possible that employees with low mental health described their organizational culture in negative terms because of their mental health status, and this may be important if a large number of employees had low mental health within a specific worksite. Second, it may be that evaluations of organizational culture are influenced by gender, age, nationality, seniority, education, and hierarchical level [52]. Women may notice some components and men other components of the culture in their organization. Older employees may notice a more traditional organizational culture and young workers may give more weight to items related to autonomy and social responsibility. In cross-cultural research, it is acknowledged that national cultures influence values, attitudes and beliefs [67-69]. Therefore, the number and types of organizational cultures may also vary from one country to another. Length of service in an organization could also influence perceptions of organizational culture as a result of the socialization process and the internalization of corporate values [14,70]. Further analysis is needed to clarify these possible confounding effects. Third, multilevel exploratory and confirmatory methods are available [71] to test within- and between-group factor models, but the sample of workplaces was too small in our study to perform these kinds of analysis with 26 items. Finally, the specific contribution of organizational culture to mental health and well-being will need to be evaluated in future studies against other workplace and individual factors also contributing to psychological distress, depression, emotional exhaustion, and well-being.

## Conclusions

In conclusion, this study provides strong support for the use of the OCP scale for measuring organizational culture in population-based occupational health research in a way that is consistent with the Competing Values Framework. It addresses the need to validate instruments that capture at least some aspects of organizational culture [72]. In particular, the Group organizational culture, because of its capacity to distinguish between workplaces and its associations with mental health and well-being outcomes, needs to be considered as a relevant factor in occupational health studies. Multilevel research and policy could benefit from these findings in efforts to provide a more complete understanding of the influence of organizational culture on business strategy, work design, employee attitudes, behavior, health and well-being.

## Appendix 1

Table 6 of Appendix 1 presents the final distribution of the 23 retained OCP items according to culture types Group, Hierarchical, Developmental, and Rational.

**Table 6:** Final distribution of the OCP items for each culture type

**Culture type**	**OCP items**
Group	Fairness (OCP1)
	Respect for the individual’s rights (OCP2)
	Tolerance (OCP3)
	Being socially responsible (OCP4)
	Being people oriented (OCP10)
	Being team oriented (OCP11)
	Working in collaboration with others (OCP12)
Hierarchical	Being careful (OCP19)
	Paying attention to detail (OCP20)
	Being precise (OCP21)
	Being rule oriented (OCP22)
	Security of employment Stability (OCP23)
	Stability (OCP24)
Developmental	Action oriented (OCP13)
	A willingness to experiment (OCP14)
	Being quick to take advantage of opportunities (OCP16)
	Being innovative (OCP17)
	Risk taking (OCP18)
Rational	Being competitive (OCP5)
	Achievement orientation (OCP6)
	Having high expectations for performance (OCP7)
	Being results oriented (OCP8)
	Being analytical (OCP9)

## Competing interests

The authors declares that they have no competing interests.

## Authors’ contributions

AM planned, collected, and analysed the data, and is lead author. VH and JDG assisted in the conceptual development, verification stages of the study, and writing (VH). All authors read and approved the final manuscript.

## Pre-publication history

The pre-publication history for this paper can be accessed here:

http://www.biomedcentral.com/1471-2458/13/443/prepub
